# Social cognition as a mediator between cognitive reserve and psychosocial functioning in patients with first episode psychosis

**DOI:** 10.1192/j.eurpsy.2021.436

**Published:** 2021-08-13

**Authors:** I. González-Ortega, S. Alberich-Mesa, E. Echeburúa, M. Bernardo, B. Cabrera, S. Amoretti, A. Lobo, C. Arango, I. Corripio, E. Vieta, E. De La Serna, R. Rodriguez-Jimenez, R. Segarra, J.M. López-Ilundain, A. Sánchez-Torres, M. Cuesta, A. González-Pinto

**Affiliations:** 1 Department Of Psychiatry, Araba University Hospital, Araba University Hospital, BIOARABA RESEARCH INSTITUTE, University OF THE BASQUE COUNTRY, CIBERSAM, Vitoria-Gasteiz, Spain; 2 Departametn Of Psychiatry, Araba University Hospital, Araba University Hospital, BIOARABA RESEARCH INSTITUTE, University OF THE BASQUE COUNTRY, CIBERSAM, Vitoria-Gasteiz, Spain; 3 Psychology, UNIVERSITY OF THE BASQUE COUNTRY, BIODONOSTIA, CIBERSAM, San Sebastián, Spain; 4 Psychiatry, Barcelona Clinic Schizophrenia Unit, Neuroscience Institute, Hospital Clinic of Barcelona, CIBERSAM, Barcelona, Spain; 5 Medicine And Psychiatry, University of Zaragoza, Aragon Institute for Health Sciences (IIS Aragón), Zaragoza, Spain; 6 Child And Adolescent Department. Institute Of Psychiatry And Mental Health, Hospital General Universitario Gregorio Marañón. CIBERSAM, Madrid, Spain; 7 Psychiatry, Institut d’Investigació Biomèdica-Sant Pau (IIB-SANT PAU), Barcelona, Spain; 8 Institute Of Neuroscience, Hospital Clinic, University Of Barcelona, Bipolar and Depressive Disorders Unit, IDIBAPS, CIBERSAM, Barcelona, Spain; 9 Child And Adolescent Psychiatry, Hospital Clinic of Barcelona, CIBERSAM, Barcelona, Spain; 10 Psychiatry, 12 de Octubre Hospital Research Institute, CIBERSAM, Madrid, Spain; 11 Neurosciences, University of the Basque Country, Cruces University Hospital, Biocruces Bizkaia Health Research Institute, CIBERSAM, Leioa, Spain; 12 Psychiatry, Navarre Hospital Complex, IdiSNA, Navarre Institute for Health Research, Pamplona, Spain

**Keywords:** first episode psychosis, social cognition, cognitive reserve, functioning

## Abstract

**Introduction:**

Social cognition has been associated with functional outcome in patients with first episode psychosis (FEP). Social cognition has also been associated with neurocognition and cognitive reserve. Although cognitive reserve, neurocognitive functioning, social cognition, and functional outcome are related, the direction of their associations is not clear.

**Objectives:**

The aim of the study was to analyze the influence of social cognition as a mediator between cognitive reserve and cognitive domains on functioning in FEP both at baseline and at 2 years.

**Methods:**

The sample of the study was composed of 282 FEP patients followed up for 2 years. To analyze whether social cognition mediates the influence of cognitive reserve and cognitive domains on functioning, a path analysis was performed. The statistical significance of any mediation effects was evaluated by bootstrap analysis.

**Results:**

At baseline, as neither cognitive reserve nor the cognitive domains studied were related to functioning, the conditions for mediation were not satisfied. Nevertheless, at 2 years of follow-up, social cognition acted as a mediator between cognitive reserve and functioning. Likewise, social cognition was a mediator between verbal memory and functional outcome. The results of the bootstrap analysis confirmed these significant mediations (95% bootstrapped CI (−10.215 to −0.337) and (−4.731 to −0.605) respectively).

**Conclusions:**

Cognitive reserve and neurocognition are related to functioning, and social cognition mediates in this relationship.

**Disclosure:**

This work was supported by the Carlos III Institute of Health and European Fund for Regional Development (PI08/1213, PI11/ 01977, PI14/01900, PI08/01026, PI11/02831, PI14/01621, PI08/1161, PI16/ 00359, PI16/01164, PI18/00805), the Basque Foundation for He

